# Polyphyllin B induces ferroptosis in triple-negative breast cancer by inactivating NRF2

**DOI:** 10.3389/fphar.2026.1803145

**Published:** 2026-05-20

**Authors:** Ping Deng, Shouhai Zhu, Yuxuan Wu, Zhiju Fang, Aini Zhang, Dongyi Zhang, Xiaoqu Hu, Xiang Zhou

**Affiliations:** 1 Department of Breast Surgery, The First Affiliated Hospital of Wenzhou Medical University, Wenzhou, China; 2 Department of Oncology, Mayo Clinic, Rochester, MN, United States; 3 Department of Burn Surgery, The First Affiliated Hospital of Wenzhou Medical University, Wenzhou, China

**Keywords:** breast cancer, ferroptosis, Nrf2, polyphyllin B, triple negative breast cancer

## Abstract

**Background:**

Triple-negative breast cancer (TNBC) is the most aggressive subtype of breast cancer (BC) and lacks effective treatment options. Polyphyllin B (PB), a saponin extracted from Paris formosana Hayata, has shown potential in cancer therapy. However, its anti-TNBC effects and mechanisms are not well studied. This study investigated the effects and underlying mechanisms of PB in TNBC.

**Methods:**

CCK8 assay, Wound healing assay, Transwell migration experiments, and clonogenic assay were performed to assess the effects of PB in three TNBC cells lines. Iron level, lipid ROS, and MDA levels were assessed as ferroptosis markers. Mechanistic studies were conducted using molecular docking, Western blot, immunofluorescence, RT-qPCR, Nrf2 overexpression assays and establishment of a TNBC mouse model.

**Results:**

PB treatment resulted in significant cell death and inhibited the migration of TNBC cells. Accumulation of iron and MDA suggested that PB induces ferroptosis. *In vitro* and *in vivo*, PB downregulated the expression of nuclear factor E2-related factor 2 (NRF2) and sequestosome-1 (P62) and upregulated the expression of Kelch-like ECH-associated protein 1 (KEAP1). Furthermore, it modulated the expression of key ferroptosis regulators, including acyl-CoA synthetase long-chain family member 4 (ACSL4) and glutathione peroxidase 4 (GPX4). In a TNBC mouse model, PB suppressed tumor growth and increased malondialdehyde accumulation.

**Conclusion:**

PB inhibits cell proliferation and migration and induces ferroptosis in TNBC through the inactivation of NRF2. PB is a promising candidate for TNBC treatment that warrants further investigation.

## Introduction

BC is one of the most prevalent malignancies among women worldwide (Li Y. et al., 2022). According to global statistics, there were about 2.3 million new breast cancer cases and 0.67 million deaths in 2022, which accounted for about 6.9% of all cancer deaths (Bray et al., 2024; Fu et al., 2025), making breast cancer the second leading cause of cancer-related deaths (Sung et al., 2021). TNBC accounts for approximately 15% of all BC cases (Mehraj et al., 2022). TNBC has high mortality and recurrence rates due to its high tumor heterogeneity (Li Y. et al., 2022; Yang et al., 2023), specific mutations, and abnormal activation of signaling pathways (Derakhshan and Reis-Filho, 2022), all of which complicate the identification of effective therapeutic targets (Li Y. et al., 2022). Currently, the primary treatments for TNBC are surgery, radiotherapy, and chemotherapy. However, these treatments cannot cure all patients, necessitating the development of new therapies.

Ferroptosis is a newly discovered cell death pattern distinct from autophagy and apoptosis (Stockwell et al., 2017) and is characterized by lipid peroxidation and iron accumulation. Morphologically, it is characterized by reduced mitochondrial volume, increased bilayer membrane density, and loss of mitochondrial cristae (Li J. et al., 2020; Dixon et al., 2012). Biochemically, ferroptosis is regulated by various factors, including GPX4 and ACSL4. GPX4 inhibits lipid peroxidation, whereas ACSL4 activates the iron-containing enzyme lipoxygenase during ferroptosis (Yan et al., 2021). Recent studies have indicated that TNBC exhibits heterogeneous phenotypes in terms of ferroptosis-related metabolites and metabolic pathways (Yang et al., 2023; Wang Z. et al., 2023). Abundant transferrin receptors on the TNBC cell surface indicate a high capacity for iron uptake. High iron levels can reprogram cellular metabolism and satisfy the rapid growth and proliferation requirements of cancer cells. GPX4 and GSH are necessary for TNBC cells to escape from oxidative stress. Based on this heterogeneity, targeting ferroptosis can be used to reverse chemotherapeutic resistance in TNBC treatments; finding new ferroptosis inducers and targeting the regulator of ferroptosis are potential therapeutic strategies in TNBC (Li et al., 2023a; Szulc and Woźniak, 2025; Jiang et al., 2021).

NRF2 plays an important role in ferroptosis by inhibiting iron accumulation and limiting ROS production (Mou et al., 2019). Normally, NRF2 is regulated by the ubiquitination and proteasomal degradation of KEAP1 (Adinolfi et al., 2023). There are conflicting perspectives regarding NRF2’s role in tumor therapy. Some research suggests activation of NRF2 is helpful for cancer treatment as it can prevent chemical carcinogen-mediated carcinogenesis. Others believe that inhibition of NRF2 is beneficial, as it can enhance chemotherapeutic efficacy in certain cancers, such as lung cancer. Recently, the effects of NRF2 have gained research interest, with the KEAP1/NRF2 signaling pathway shown to inhibit ferroptosis in several cancer types (Ren et al., 2011; Chowdhry et al., 2013; Lien et al., 2016).

PB, a saponin isolated from *Paris formosana* Hayata (Wu et al., 1990), has been previously demonstrated to exert anti-tumor effects in lung cancer (Li et al., 2023b), gastric cancer (Hu et al., 2023), and hepatocellular carcinoma (Lin et al., 2020). In this study, we demonstrated that PB is an effective natural product extract against TNBC. We further investigated its potential mechanism in TNBC, revealing that PB serves as a novel ferroptosis inducer by inhibiting NRF2 and GPX4. These results provide a theoretical basis for the future clinical application of PB in treating TNBC.

## Materials and methods

### Reagents and antibodies

PB (CAS: 50773-42-7, purity: >=98%) was purchased from ChemFaces (Wuhan, Hubei, China). Fer-1(CAS: 347174-05-4, purity: 99.71%) was purchased from MedChemExpress (MCE) (Shanghai, China).

### Cell culture

HS-578T, MDA-MB-231, and BT-549 cells were obtained from the Cell Bank of the Shanghai Institute of Biochemistry and Cell Biology (Shanghai, China). HS-578T and MDA-MB-231 cells were cultured in Dulbecco’s Modified Eagle’s Medium (DMEM) (Gibco, Germany) containing 10% fetal bovine serum (FBS) (Thermo Fisher Scientific Inc, USA) in a humidified atmosphere with 5% CO_2_ at 37 °C. BT-549 cells were cultured in RPMI-1640 (Gibco, Germany) containing 10% FBS in a humidified atmosphere with 5% CO_2_ at 37 °C.

### Cell viability assay

Approximately 5000 cells were seeded into 96-well plates and incubated overnight. Subsequently, the TNBC cells were treated with increasing concentrations of PB (0.625, 1.0, 1.25, 2, 2.5, 3.0, 5.0 μM) for 24 h or 48 h. Cell Counting Kit-8 (CCK8) (Dojindo, Japan) reagent (10 µL/well) was added to the plates and then incubated at 37 °C for 2 h. Absorbance was then measured at 450 nm. Cell viability was assessed using GraphPad Prism 9.0.

### Wound healing assay

TNBC cells were seeded into 6-well plates. When the cells had grown to 80%–90% confluence, a sterile 10 μL pipette tip was used to scrape the cell monolayer and create linear scratch wound. Then, the wells were washed with PBS to remove detached cells from the plates. Cells were cultured in serum-free medium with or without PB (0.5 µM) for 48 h. Images were taken at 0 h, 24 h, and 48 h to track cell migration into the wound area using a microscope (Leica, Wetzlar, Germany).

### Transwell migration experiments

Approximately 3 × 10^4^ cells were suspended in 200 µL of serum-free medium and seeded into the upper chambers of the Transwell plates, treated with or without PB (0.5 µM). 500 μL of complete medium containing 20% FBS was added to the lower chambers of Transwell plates. After 48 h, the cells on the upper surface of the membrane were removed with sterile cotton swabs and washed with PBS. Then the membranes were stained with crystal violet and analyzed under a microscope (Leica, Wetzlar, Germany).

### Colony assay

Approximately 1000 cells were seeded into 6-well plates and treated with PB (0.5 µM), either alone or in combination with Fer-1 (1 μM), for 6–14 days. After the treatment period, the cells were fixed with 4% paraformaldehyde, stained with crystal violet (Biosharp, China) for 20 min, and then air-dried.

### Measurement of iron contents

The iron concentration in TNBC cells was measured using the Iron Colorimetric Assay Kit (ab83366, Abcam) according to the manufacturer’s protocol. Briefly, approximately 2 × 10^6^ cells were rapidly homogenized in iron assay buffer on ice. After centrifugation, the supernatant was collected and mixed with assay buffer, then incubated for 30 min at room temperature. Next, 100 μL of iron probe was added, mixed and incubated at 37 °C for 60 min in the dark. Finally, the absorbance was measured at 593 nm using a microplate reader.

### Western blot

Proteins from tumor tissues and cells were extracted using Radio-Immunoprecipitation Assay buffer (RIPA, Beyotime), separated on 10% or 12.5% sodium dodecyl sulfate-polyacrylamide gels, and transferred to polyvinylidene fluoride (PVDF) membranes (Millipore, United States). Membranes then were blocked with 5% skim milk at room temperature for 2 h. They were then incubated with primary antibodies overnight at 4 °C, then washed three times with Tris-buffered saline containing 0.5% Tween-20 (TBST), incubated with secondary antibodies for 1 h at room temperature the following day. The primary antibodies used in Western blot assays include anti-P62 (18420-1-AP, Proteintech), anti-KEAP1 (10503-2-AP, Proteintech), anti-NRF2(16396-1-AP, Proteintech), anti-ACSL4 (EPR8640, Abcam), anti-GPX4 (67763-1-Ig, Proteintech) and anti-GAPDH (10494-1-AP, Proteintech), the secondary antibodies include HRP-conjugated goat anti-mouse IgG (H + L) (SA00001-1, Proteintech) and HRP-conjugated goat anti-rabbit IgG (H + L) (SA00001-2, Proteintech). Immunoreactive bands were visualized using an enhanced chemiluminescence (ECL) detection system, and densitometric quantification was performed using ImageJ software.

### Real-time quantitative PCR

RNA from TNBC cells was extracted with TRIzol and reverse-transcribed into cDNA. RT-qPCR was performed using Taq polymerase (TAKARA, Japan). GAPDH was used as the reference gene for normalization, and the relative expression levels of target genes were calculated using the ΔΔCq method. The primer sequences (5′→3′) for the target genes, bought from Sangon Biotech (Shanghai, China), are as follows: 5′-TGA​TTG​AGT​CCC​TCT​CCC​AGA​TGC-3′ (P62,F) and 5′-GCC​GCT​CCG​ATG​TCA​TAG​TTC​TTG-3′ (P62,R); 5′-CCTGGACAGTGTGGAGTGTTACG-3′(Keap1,F) and 5′-AGTTCTGCTGGTCAATCTGCTTCC-3′(Keap1,R); 5′-AGTCCAGAAGCCAAACTGACAGAAG-3′(NRF2,F) and 5′-GGAGAGGATGCTGCTGAAGGAATC-3′(NRF2, R); 5′-GGTCGGAGTCAACGGATTTG-3′(GAPDH,F) and 5′-ATGAGCCCCAGCCTTCTCCAT-3′(GAPDH,R).

### Measurement of lipid ROS

To investigate ROS generation, approximately 5 × 10^4^ cells were seeded in 6-well plates and treated with PB (0.5 μM), either alone or in combination with Fer-1 (1 μM) for 48 h. Subsequently, these cells were incubated with 10 µM DCFH-DA (S0033, Beyotime) for 20 min at 37 °C in a light-free environment. Finally, images were captured using a fluorescence microscope.

### Molecular docking and bioinformatic analysis

Molecular docking was performed using Auto Dock Vina software. The crystal structure of GPX4 and NRF2 were retrieved from the Research Collaboratory for Structural Bioinformatics Protein Data Bank (PDB, http://www.rcsb.org/pdb/). The PB structure was obtained from the PubChem Compound Database (https://pubchem.ncbi.nlm.nih.gov/). The best-fit complexes were visualized and analyzed using PyMOL 2.4.0.

The mRNA expression levels of GPX4 and NRF2 in tumors and adjacent normal tissues across a pan-cancer dataset were analyzed via the TIMER database (TIMER1.0, https://compbio.cn/timer1/), Kaplan-Meier survival analysis of NRF2 expression in BC was conducted using data from the TCGA database.

### Cell transfection

MDA-MB-231 cells were transfected with an NFE2L2 (NRF2) overexpression plasmid (Gene Chem, Shanghai) using Lipofectamine™ 2000 (Invitrogen, United States) transiently, according to the manufacturer’s instructions. After 24 h, the efficiency of overexpression was confirmed via western blotting and immunofluorescence analysis.

### Immunofluorescence staining

TNBC cells were seeded on glass slides in 24-well plates and treated with PB (0.5 μM), either alone or in combination with Fer-1 (1 μM) for 48 h. Subsequently, the cells were fixed with 4% paraformaldehyde and permeabilized with 0.1% Triton X-100 (Solarbio, China), followed by blocking with goat serum (BOSTER, China). Next, the cells were incubated with primary antibodies overnight and then incubated with secondary antibodies for 1 h. DAPI was used to visualize the nuclei. After sealing with an anti-fluorescence quencher, the slides were imaged by fluorescence microscope (Leica, Germany).

### Animal experiment protocol

Four-week-old female BALB/c-nu nude mice were obtained from the Laboratory Animal Center of Wenzhou Medical University (Wenzhou, China), and the study was approved by the ethics committee of Wenzhou Medical University (WYYY-AEC-YS-2024-0534). A total of 100 μL PBS containing 2 × 10^6^ MDA-MB-231 cells was injected subcutaneously into mice. When the tumor volume reached approximately 50 mm^3^, the mice were randomly divided into three experimental groups (five mice per group) with similar mean body weights and tumor volumes: a PBS control group, a 2 mg/kg PB treatment group, and a 2 mg/kg PB + 1 mg/kg Fer-1 combination treatment group, followed by intraperitoneal injection of the corresponding treatments every other day for 2 weeks. The tumor size and body weight of the mice were recorded every other day throughout the study period. Finally, the mice were euthanized by cervical dislocation, and the tumors were collected for histological and western blot analyses.

### Measurement of malondialdehyde (MDA) levels

Lipid peroxidation in tumor tissues was assessed by measuring the concentration of MDA using an MDA Assay Kit (S0131, Beyotime) in accordance with the manufacturer’s instructions.

### Hematoxylin and eosin (HE) staining and immunohistochemistry (IHC)

All experiments were repeated independently at least three times. All data were expressed as the mean ± standard deviation (SD). Statistical comparisons between two or more groups were performed using Kruskal–Wallis analysis of variance and multifactorial ANOVA, while other analyses utilized the t-test. A P-value of <0.05 was considered statistically significant. GraphPad Prism 8.0 (San Diego, CA, USA) was used for all statistical analyses.

### Statistical analysis

All experiments were repeated independently at least three times. All data were expressed as the mean ± standard deviation (SD). Statistical comparisons between two or more groups were performed using Kruskal–Wallis analysis of variance and multifactorial ANOVA, while other analyses utilized the t-test. A P-value of <0.05 was considered statistically significant. GraphPad Prism 8.0 (San Diego, CA, USA) was used for all statistical analyses.

## Results

### PB inhibited the proliferation of TNBC cells *in vitro*

**FIGURE 1:**
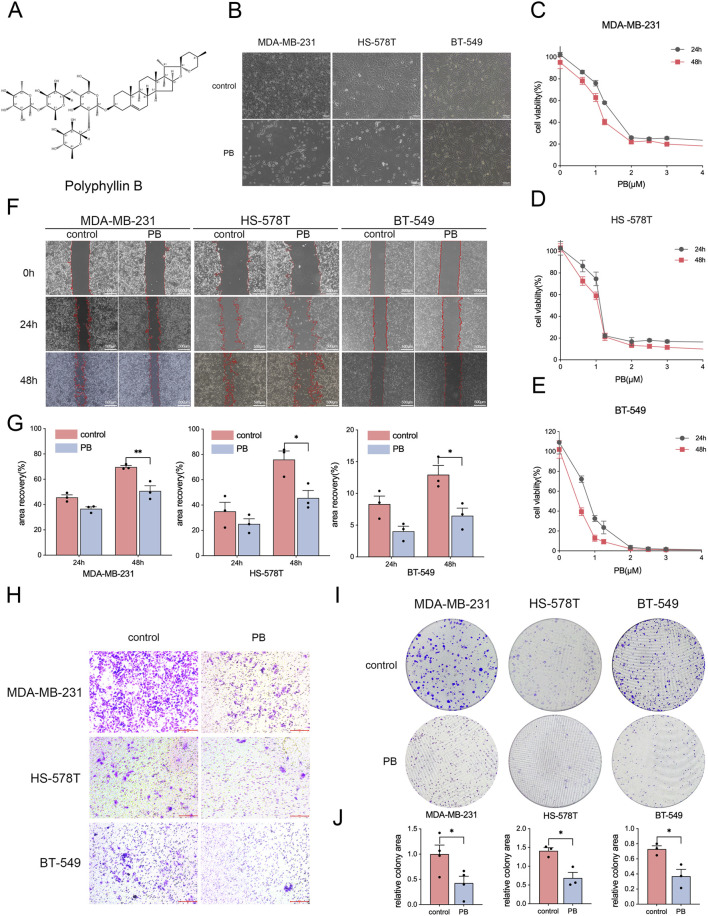
PB inhibits the proliferation of TNBC cells *in vitro*. **(A)** Chemical structure of PB. **(B)** Phase-contrast microscopic images of TNBC cells with or without PB treatment (magnification, 10×). **(C–E)** The effects of PB on the proliferation of TNBC cells were determined through CCK8 assays. **(F,G)** Wound healing assays of TNBC cells with or without PB treatment, followed by quantitative analysis. **(H)** Transwell assays of TNBC cells with or without PB treatment. Bar = 200 μm. **(I,J)** Colony assays of TNBC cells with or without PB treatment, followed by quantitative analysis. *P < 0.05, **P < 0.01.

Several experiments were conducted to assess the effects of PB (Figure 1A), on TNBC cells. Phase-contrast microscopy revealed noticeable morphological changes in cells, such as cell rounding and decreased cell numbers (Figure 1B), which suggested inhibition of cell viability by PB. CCK8 assays revealed that PB significantly inhibited cell proliferation in a dose-dependent manner with the 48-h treatment being more effective. The 48-h IC50 values for PB were determined to be 1.183 μM in MDA-MB-231 cells, 1.009 μM in HS-578T cells, and 0.5371 μM in BT-549 cells (Figures 1C–E). Wound healing and Transwell migration assays showed that PB inhibited the migration capacity of TNBC cells (Figures 1F–H), and colony assays showed it also suppressed colony formation in these cells (Figures 1I,J). Collectively, these findings demonstrated that PB effectively inhibited the growth and survival of TNBC cells *in vitro*.

### PB induced ferroptosis *in vitro*

**FIGURE 2:**
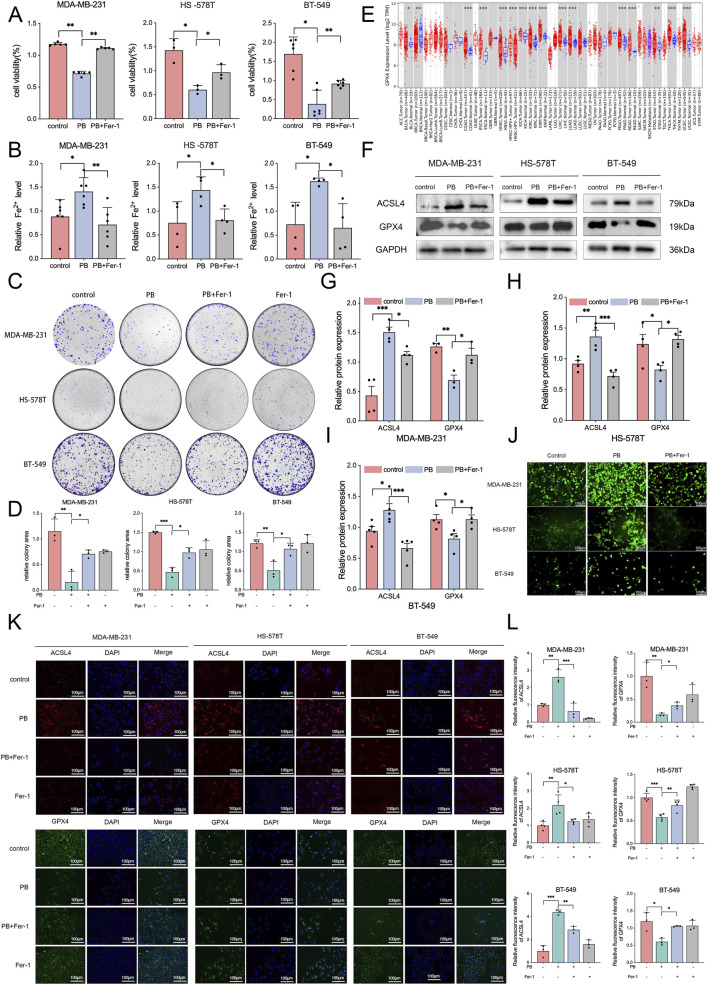
PB induces ferroptosis *in vitro*. **(A)** Viability of TNBC cells treated with PB or in combination with Fer-1 for 48 h, assessed via CCK8 assay. **(B)** Measurement of iron levels to evaluate the iron accumulation in TNBC cells. **(C,D)** Proliferative ability of TNBC cells treated with PB or in combination with Fer-1 for 48 h, assessed via Colony assays. **(E)** TIMER database online analysis results of GPX4. **(F–I)** Protein expression of ACSL4 and GPX4 in TNBC cells. **(J)** ROS levels in TNBC cells. **(K,L)** Immunofluorescence analysis of ACSL4 and GPX4 in TNBC cells. *P < 0.05, **P < 0.01, ***P < 0.001.

TNBC is particularly susceptible to ferroptosis and ferroptosis has been demonstrated to be a potential therapeutic target for TNBC (Verma et al., 2020). Ferroptosis is a type of cell death characterized by iron dependence and lipid peroxidation. To determine whether PB induces ferroptosis, TNBC cells were treated with PB alone or in combination with the ferroptosis inhibitor Fer-1 for 48 h. CCK8 assays showed that the anti-proliferative effects of PB were rescued by Fer-1 (Figure 2A). Additionally, PB induced iron accumulation and this accumulation was further reduced by Fer-1 (Figure 2B). In colony assays, Fer-1 reduced the PB-mediated inhibition of colony formation ability (Figures 2C,D). Taken together, these results demonstrate that PB treatment triggers ferroptosis in TNBC cells.

Ferroptosis is regulated by GPX4 and ACSL4 (Glibetic and Weichhaus, 2025). GPX4 is a central inhibitor of ferroptosis, collaborates with glutathione to covert lipid peroxidation and inhibit ROS accumulation indirectly (Bersuker et al., 2019). Our analysis of the TIMER database revealed a difference in GPX4 mRNA expression between breast tumors and adjacent normal tissues (Figure 2E). When GPX4 is in inactivated or absent, ACSL4 become indispensable for lipid peroxidation in ferroptosis (He et al., 2022). Therefore, we examined the protein expression and fluorescence intensities of GPX4 and ACSL4 in TNBC cells. The results showed that PB effectively decreased GPX4 protein expression and elevated ACSL4 protein expression in TNBC cells (Figures 2F–I). With substrates provided by ACSL4 and loss of the quenching function of GPX4, ROS production increased through the iron-mediated Fenton reaction, as expected (Figure 2J). Changes in the fluorescence intensities of GPX4 and ACSL4 correlated with changes in their protein expression levels (Figures 2K,L). These effects were partly reversed by Fer-1. In conclusion, our findings indicate that the anticancer effect of PB is partly attributable to ferroptosis in TNBC cells, a process mediated by GPX4 and ACSL4.

### PB suppressed TNBC cells by inactivating NRF2

Studies have demonstrated that total saponins extracted from *Rhizoma Paridis* can alleviate oxidative stress injury by upregulating the NRF2 pathway, but it remains unclear which specific saponins mediate this effect and what the underlying mechanism is (Zhao et al., 2020). The antioxidant, iron, and intermediate metabolic statuses of cells are inseparable from the regulation by NRF2, which has been shown to be a critical regulator of ferroptosis and GPX4 (Dodson et al., 2019).

**FIGURE 3:**
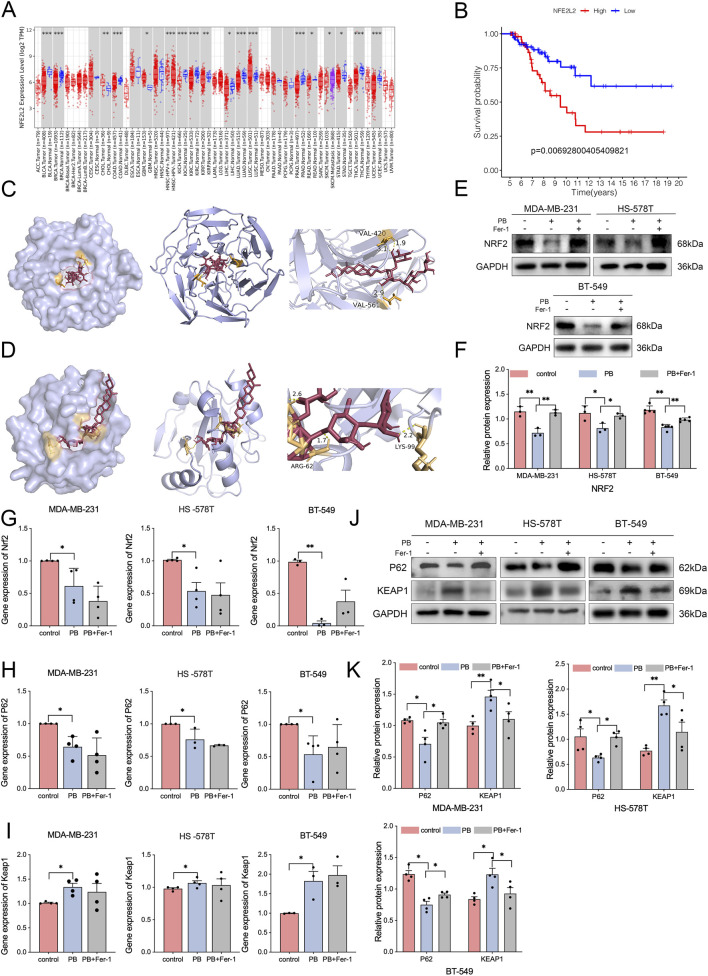
PB induces ferroptosis by inactivating NRF2. **(A)** TIMER database online analysis results of NRF2. **(B)** Kaplan-Meier survival curve of NRF2 in BC based on TCGA database. **(C,D)** Molecular docking results between PB and NRF2 with GPX4. **(E–G)** The gene and protein expression of NRF2 in TNBC cells, followed by quantitative analysis. **(H,I)** The gene expression of P62 and KEAP1. **(J,K)** The protein expression of P62 and KEAP1, followed by quantitative analysis. *P < 0.05, **P < 0.01.

Through TIMER database analysis, we focused on the differences in NRF2 mRNA expression between tumors and adjacent normal tissues (Figure 3A), so we further explored the effects of NRF2 on BC survival. Using TCGA clinical data with a survival period of more than 5 years, we found that low NRF2 expression is associated with the long-term survival of patients (Figure 3B). Molecular docking assays revealed the binding sites each for PB with NRF2 and with GPX4, and the binding energies were measured as −11.8 kcal/mol and −8.7 kcal/mol, respectively (Figures 3C,D). Based on the higher binding energies, we indicated NRF2 as the primary binding partner of PB. Next, we examined the gene and protein expression of NRF2 in TNBC cells, the results showed that PB dramatically reduced both the protein and gene expression of NRF2 (Figures 3E–G), while Fer-1 only reversed the decrease in NRF2 protein expression. These findings suggest that PB acts on TNBC cells partly relay on NRF2.

As NRF2 expression is commonly regulated by P62 and KEAP1 (Li T. et al., 2020; Liu et al., 2023), we further examined the protein and gene expression of P62 and KEAP1.These results showed that PB decreased both the gene and protein expression of P62, while it increased those of KEAP1. In contrast, Fer-1 reversed the protein expression without affecting the gene expression of either P62 or KEAP1 (Figures 3H–K). Therefore, we further conclude that PB-resulted ferroptosis via the NRF2 pathway is modulated by P62 and KEAP1.

### NRF2 negatively regulated ferroptosis induced by PB

**FIGURE 4:**
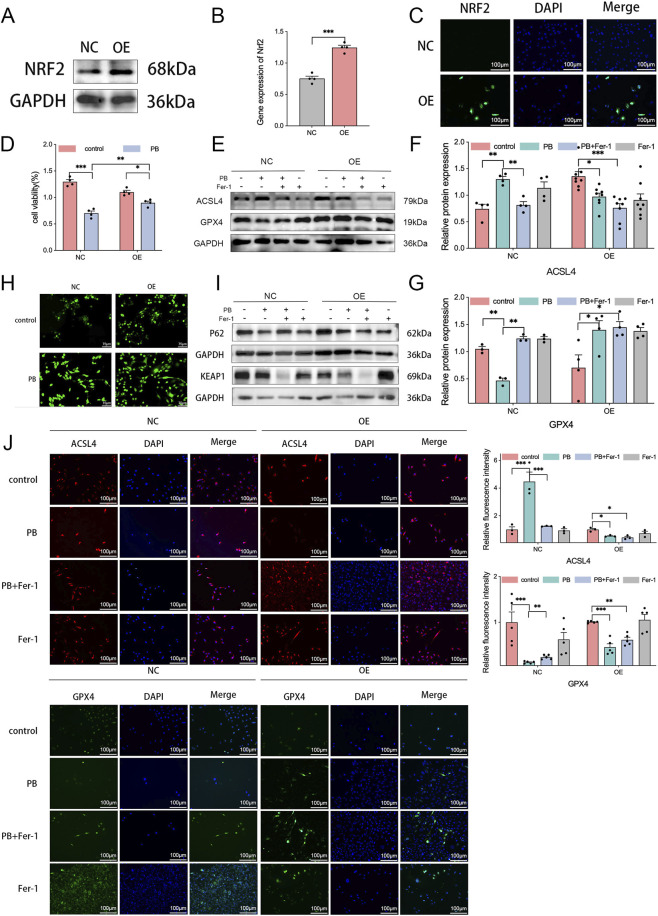
NRF2 inhibits PB-induced ferroptosis. **(A–C)** Relative protein expression was measured by western blotting and immunofluorescence in NRF2-overexpressing cells, followed by quantitative analysis. *P < 0.05, **P < 0.01, ***P < 0.001. **(D)** CCK8 assays in NRF2-overexpressing cells. **(E–G)** Protein expression of ACSL4, and GPX4 in NRF2-overexpressing cells, followed by quantitative analysis. **(H)** ROS levels in NRF2-overexpressing cells. **(I)** Protein expression of P62, and KEAP1 in NRF2-overexpressing cells. **(J)** Immunofluorescence analysis of ACSL4 and GPX4 in NRF2-overexpressing cells.

We established NRF2-overexpression MDA-MB-231 cells to investigate the effects of NRF2 on cell proliferation and ROS production in TNBC (Figures 4A–C). The CCK8 results revealed that overexpression of NRF2 partially counteracted the inhibitory effect of PB on cell proliferation (Figure 4D). With the treatment of PB, GPX4 protein expression was not decreased but increased, while ACSL4 protein expression decreased in NRF2-overexpression cells (Figures 4E–G). These protein expression changes were further verified by immunofluorescence staining (Figure 4J). Furthermore, overexpression of NRF2 partly suppressed the production of ROS induced by PB (Figure 4H). However, in the NRF2-overexpressing cells, P62 and KEAP1 protein expression were not significantly affected by PB, compared with the protein expression changes in cells transfected with an empty vector (Figure 4I). These results suggested that PB induces ferroptosis through NRF2, with GPX4 and ACSL4 acting as downstream effectors, while P62 and KEAP1 likely act as upstream effectors.

### PB suppressed tumor growth and induced ferroptosis *in vivo*

**FIGURE 5:**
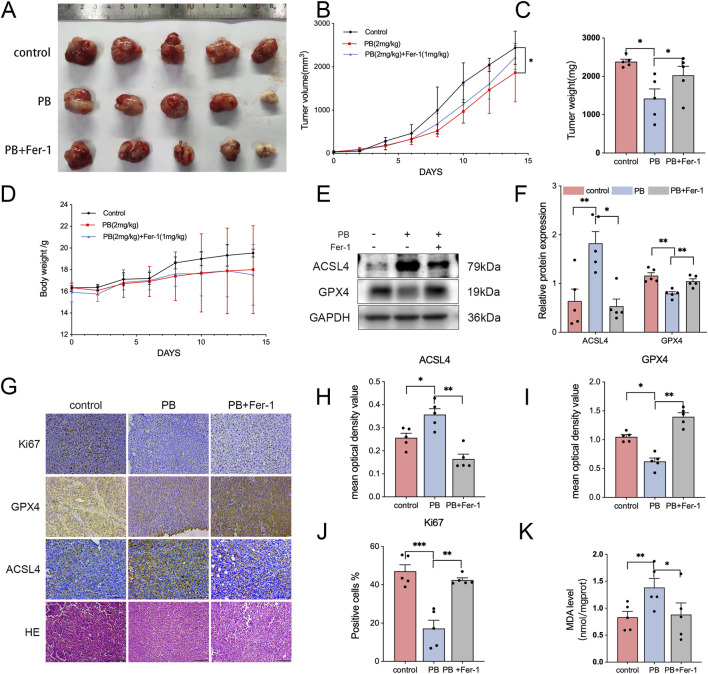
PB induces ferroptosis *in vivo*. **(A)** Photographs of tumors. **(B)** Tumor volumes and **(C)** Tumor weights. The data are presented as means ± SDs, with n = 5. **(D)** Mice weight. **(E,F)** The protein expression of ACSL4 and GPX4 in subcutaneous xenografts, followed by quantitative analysis. **(G–J)** Immunohistochemical staining of tumor sections for the cell proliferation marker Ki-67, ferroptosis markers ACSL4 and GPX4, and Hematoxylin and Eosin (H&E), followed by quantitative analysis. Bar = 100 μm **(K)** The MDA level in subcutaneous xenografts. *P < 0.05, **P < 0.01, ***P < 0.001.

To evaluate the antitumor activity of PB *in vivo*, we established a mouse xenograft tumor model using MDA-MB-231 cells. Tumor imaging and measurements of tumor weight and volume revealed that xenograft tumors in PB-treated mice grew more slowly than those in PBS-treated mice (Figures 5A–C). Throughout the experiment, there were no abnormalities in eating or behavioral activities were observed in any of the groups (PBS, 2 mg/kg PB, and 2 mg/kg PB + 1 mg/kg Fer-1). Although body weight increased in both the PB-treated and combination-treated groups, the rate of body weight gain was slower than that in the control group (Figure 5D).

**FIGURE 6:**
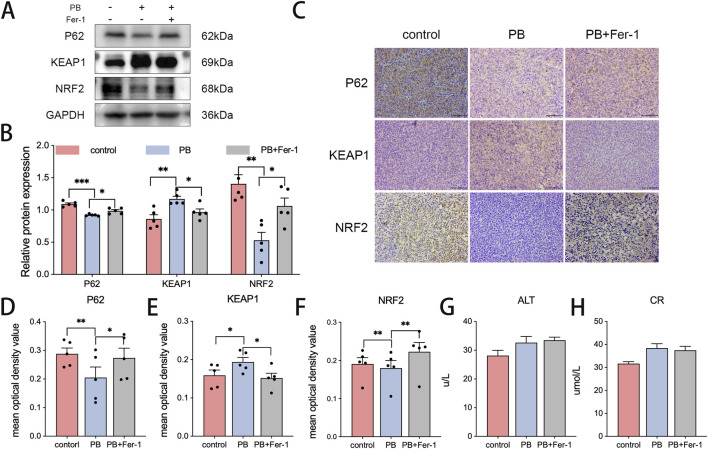
PB induces ferroptosis through NRF2 *in vivo*. **(A,B)** The protein expression of P62, KEAP1, and NRF2 in subcutaneous xenografts, followed by quantitative analysis. **(C)** Immunohistochemical staining of tumor sections for P62, KEAP1, and NRF2, followed by quantitative analysis. Bar = 100 μm *P < 0.05, **P < 0.01 **(D–F)**. **(G,H)** The level of ALT and CR in peripheral blood of mice model.

Ki67 staining was consistently weaker in the PB-treated group than in the control group (Figure 5G). Fer-1 reversed this reduction. Similarly, PB treatment significantly decreased GPX4 expression but increased ACSL4 expression (Figures 5E–J). The level of MDA was also reduced in the PB-treated group (Figure 5K). These results demonstrate that PB inhibited the proliferation of MDA-MB-231 cells and induced ferroptosis *in vivo*. Western blotting and immunohistochemical results revealed that PB treatment reduced P62 and NRF2 levels and elevated KEAP1 levels (Figures 6A–F), which suggests that PB exerts its antitumor effect by inhibiting NRF2, a mechanism that may be regulated by P62 and KEAP1. The levels of ALT and CR in peripheral blood suggested that PB did not cause significant host toxicity during the treatment period at this effective dose (Figures 6G,H).

## Discussion

TNBC has the worst prognosis among all BC subtypes. Despite the development of emerging immunotherapies in recent years, their efficacy is still limited to a subset of patients and exists resistance (Zhu et al., 2021). Therefore, the identification of novel molecular targets and therapeutic agents is crucial. *Rhizoma Paridis* is a traditional Chinese medicine known for its heat-clearing and detoxification properties (Li J. K. et al., 2022; Zhou et al., 2023). Previous studies have demonstrated the anticancer properties of steroidal saponin isolated from *Rhizoma Paridis* (Wang Y. et al., 2023; Yang et al., 2024). For example, polyphyllin I causes cell cycle arrest, ultimately leading to cancer cell death (Yu et al., 2018); Polyphyllin II triggers autophagy in colorectal cancer cells (Li J. K. et al., 2022); and Polyphyllin G induces both apoptosis and autophagy in human nasopharyngeal cancer cells (C et al., 2016). However, the specific mechanism of PB in TNBC remains unclear.

**FIGURE 7:**
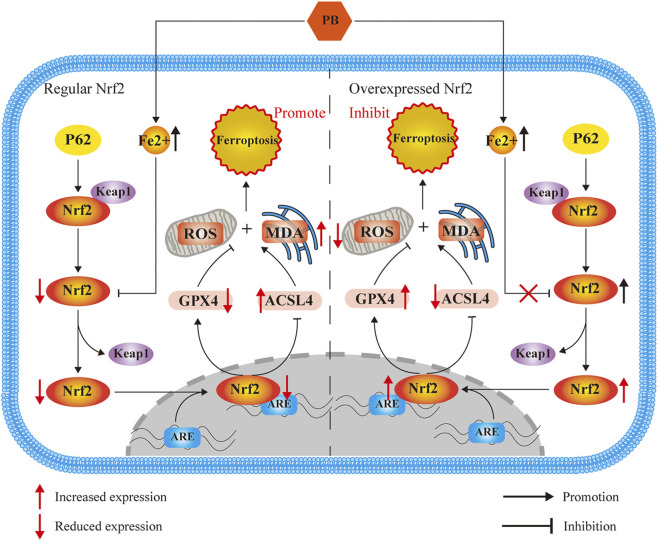
Mechanism.

Here, we observed that PB inhibited the proliferation and migration capacities of TNBC cells *in vitro*, induced cell death, and suppressed tumor growth *in vivo*. All these effects were associated with ferroptosis. Mechanistically, we demonstrated that PB induced ferroptosis via NRF2 downregulation, leading to reduced GPX4 expression and the loss of GPX4 quenching function to lipid peroxidation. Concurrently, increased ACSL4 expression and iron accumulation elevated ROS and MDA production, ultimately triggering ferroptosis in TNBC (Figure 7).

Ferroptosis is a new form of programmed cell death characterized by iron-dependent lipid peroxidation (Li J. et al., 2020). It can enhance immune responses and help overcome immunotherapy resistance to cancer (Liu et al., 2026), as well as cause cancer cells death by resulting in accumulation of lipid peroxides and iron, it can also reshape the immune microenvironment and alter the susceptibility of tumor cells to ferroptosis (Chen et al., 2026; Jia et al., 2026; Xu et al., 2022). Ferroptosis has demonstrated anticancer properties in various cancers, particularly in TNBC. TNBC has high iron levels and low antioxidant capacity, which make it susceptible to ferroptosis (Tan et al., 2025). Interestingly, in this study, PB increased ROS production and iron accumulation in TNBC cells, and elevated MDA levels in a TNBC mouse model. Importantly, all these effects were reversed by Fer-1, indicating that PB can induce ferroptosis and cause cell death in TNBC.

GPX4 and ACSL4 are key marker proteins of ferroptosis. Previous studies have shown that ACSL4 and GPX4 double KO cells exhibit stronger proliferative capacity than normal cells, implicating an important functional interplay between GPX4 and ACSL4 (Doll et al., 2017). As a central repressor of ferroptosis, GPX4 influences lipid and amino acid metabolism, as well as processes such as cell aging, oncogenesis, and cell death. GPX4 can convert the toxic lipid peroxidation and then mitigate cellular damage from ferroptosis. In the condition of GPX4 inhibition or absence, ACSL4 catalyzes the synthesis of long-chain polyunsaturated-CoAs, triggering phospholipid peroxidation and supplying substrates for ferroptosis (Qiu et al., 2024; Chen et al., 2021a; Zefrei et al., 2024). In our study, PB treatment downregulated both the gene and protein expression of GPX4 *in vitro* and *in vivo*, without GPX4 activity and with enhanced ACSL4 catalytic function, lipid peroxidation and ROS production increased accordingly, leading to ferroptosis in TNBC cells. Therefore, we propose that PB functions as an inhibitor of GPX4 in ferroptosis and its effect may not be limited to TNBC based on previous research (Hu et al., 2023).

NRF2 is a member of the cap ‘n’ collar family of basic leucine zipper transcription factors (Chen et al., 2021b), Previous studies have proofed NRF2 as a central regulator of ferroptosis and oxidative stress responses (Sun et al., 2016; Tunc et al., 2025), GPX4 transcription need NRF2 migrates to the nucleus and its initiation (Cheng et al., 2021). NRF2 role in breast cancer presents two opposing perspectives. Some researchers propose that NRF2 protects breast cancer cells by resisting oxidative stress, while others argue that it promotes cell death by enhancing it (Wang Z. et al., 2023; Luo et al., 2024). In PB-induced ferroptosis, we observed a significant decrease in NRF2 expression. By establishing an NRF2 overexpression model, we found that NRF2 overexpression inhibited PB-induced ferroptosis, decreased ACSL4 expression, and increased GPX4 expression, resulting in reduced ROS accumulation. Integrating these findings with existing studies, we conclude that NRF2 acts as a ferroptosis inhibitor that counteracts the anticancer activity of PB in TNBC.

The activity of NRF2 is primarily controlled by its endogenous inhibitor, KEAP1 (Gelerstein-Claro et al., 2025). In our study, KEAP1 expression increased and P62 expression decreased as expected following PB treatment. These changes were consistent with NRF2 decreased expression but were not significantly affected by NRF2 overexpression. So, we propose that NRF2 functions as an inhibitor of ferroptosis in PB treatment, with P62 and KEAP1 maybe the upstream regulatory factors of NRF2. However, the specific mechanisms by which P62 and KEAP1 interact to regulate NRF2 degradation following PB exposure remain unclear and warrant further investigation, representing a limitation of this study.

This study uncovers a novel anti-tumor mechanism of PB in TNBC. PB downregulates NRF2 activity, leading to reduced expression of GPX4 and increased expression of ACSl4, with iron accumulation. These changes collectively promote lipid peroxidation, culminating in ferroptosis and thereby exerting anticancer effects. We validated that PB induces TNBC cell death *in vitro* and suppresses tumor growth *in vivo*. However, several questions remain. The functional interplay between the observed changes in P62 and KEAP1 expression, as well as the specifics of NRF2 nuclear translocation and GPX4 activation, require further experimental validation. As cell death is a complex process influenced by many factors, for example, recent studies have indicated the special role of MDM2 (Wang et al., 2025; Todoric et al., 2017) and FOXC2 (Zhao et al., 2026) in TNBC. Whether any other signaling pathway or regulator contributes to PB-induced TNBC cell death also needs deep exploration. What’s more, while the overall trends were consistent, differences observed among the three TNBC cell lines merit future investigation to explain their variable responses.

## Conclusion

In conclusion, our findings provide compelling evidence that PB suppresses TNBC progression by inducing ferroptosis through NRF2 inhibition, a process mediated through the regulation of its downstream effectors, GPX4 and ACSL4. The high therapeutic efficacy and low toxicity observed in mouse models suggest that PB is a promising candidate for TNBC therapy. Further investigation of the therapeutic effects of PB in clinical trials is warranted.

## Data Availability

The raw data supporting the conclusions of this article will be made available by the authors, without undue reservation.
